# The Relation of Mild Types of Diphtheria to the Public Health

**Published:** 1903-12-26

**Authors:** 


### THE RELATION OF MILD TYPES OF DIPHTHERIA TO THE PUBLIC HEALTH.

ALTHOUGH diphtheria has lost many of its terrors and can now no longer be called "the most dreaded disease of childhood," yet its victims are still numbered by thousands, and it is really a matter of great moment to consider by what means the prevalence of the disease can be diminished. One weak point in our present defensive methods by notification and isolation lies in the fact that we do not notify and isolate the mild cases, which therefore are at liberty to walk abroad and disseminate infection. Dr. Thomas W. Salmon [1] in discussing this problem as it presents itself in New York State, where the conditions are somewhat similar to those which obtain in this country, urges the importance of recognising that diphtheria may occur in a mild form without the formation of membrane, and insists on the necessity for isolating these mild forms as well as those which are more severe. He remarks that it is hardly possible to state where the earliest record can be found of the fact that during epidemics of diphtheria there is usually an increase in the prevalence of "simple" sore throats, while the literature for many years shows incessant repetitions of the statement. Indeed most physicians who have had outbreaks of diphtheria in their practices can recall instances where mothers and nurses of sick children have been affected with this form of so-called simple sore throat. It is most essential that these cases of sore throat should receive official recognition and be assigned a definite place among the varieties of diphtheria.

It has been pointed out that a number of different conditions may follow the infection of an individual with the bacillus of diphtheria. (1) The disease which is commonly recognised as diphtheria, with the presence of membrane and the evidences of systemic poisoning, results from the infection of a susceptible person with virulent diphtheria bacilli. (2) When insusceptible individuals become infected with virulent diphtheria bacilli no lesions result, but, as is well known, the bacilli may be transmitted to susceptible persons and cause the disease in a severe form. (3) Susceptible subjects may become infected with diphtheria bacilli wholly lacking in virulence. No lesions follow this occurrence, and the spread of the disease is probably not aided by such means, for it has been shown that non-virulent bacilli do not regain virulence readily, if at all. (4) Relatively insusceptible patients may become infected with virulent diphtheria bacilli. (5) Susceptible persons may become infected with feebly virulent bacilli. Of these five possibilities No. (1) alone is sufficiently provided for by our public health laws. No. (3) perhaps needs no especial provision, for the reason mentioned above. But the remaining alternatives, which are manifested as the "simple" sore throats so prevalent during epidemics of diphtheria, call for prominent attention and prompt action. The first step would naturally be to provide a suitable name in accordance with their etiology, and Dr. Salmon suggests the name of "catarrhal diphtheria" for this form of sore throat.

He then passes on to a consideration of the diagnosis and management of these mild cases of diphtheria. The diagnosis of catarrhal diphtheria depends entirely upon bacteriological examinations. Care should therefore be taken to have such an examination carried out in every case where there is reason to suspect the possibility of diphtheritic infection, and means should be provided by the public to facilitate this custom. With regard to the management, the immediate concern is to educate the laity to a proper appreciation of the serious importance and danger to others of catarrhal diphtheria. The next step would be to include the condition in the list of notifiable diseases under its own name, and to extend to it the same sanitary regulations which relate to the management of the well marked types of diphtheria.

[1] Medical News, Nov. 21.

